# Automated Tubule Nuclei Quantification and Correlation with Oncotype DX risk categories in ER+ Breast Cancer Whole Slide Images

**DOI:** 10.1038/srep32706

**Published:** 2016-09-07

**Authors:** David Romo-Bucheli, Andrew Janowczyk, Hannah Gilmore, Eduardo Romero, Anant Madabhushi

**Affiliations:** 1 Universidad Nacional de Colombia, Engineering Faculty, Bogotá D.C, Colombia; 2 Case Western Reserve University, Biomedical Engineering department, Cleveland, OH, USA; 3 University Hospitals, School of Medicine, Cleveland, OH, USA

## Abstract

Early stage estrogen receptor positive (ER+) breast cancer (BCa) treatment is based on the presumed aggressiveness and likelihood of cancer recurrence. Oncotype DX (ODX) and other gene expression tests have allowed for distinguishing the more aggressive ER+ BCa requiring adjuvant chemotherapy from the less aggressive cancers benefiting from hormonal therapy alone. However these tests are expensive, tissue destructive and require specialized facilities. Interestingly BCa grade has been shown to be correlated with the ODX risk score. Unfortunately Bloom-Richardson (BR) grade determined by pathologists can be variable. A constituent category in BR grading is tubule formation. This study aims to develop a deep learning classifier to automatically identify tubule nuclei from whole slide images (WSI) of ER+ BCa, the hypothesis being that the ratio of tubule nuclei to overall number of nuclei (a tubule formation indicator - TFI) correlates with the corresponding ODX risk categories. This correlation was assessed in 7513 fields extracted from 174 WSI. The results suggests that low ODX/BR cases have a larger TFI than high ODX/BR cases (*p* < 0.01). The low ODX/BR cases also presented a larger TFI than that obtained for the rest of cases (*p* < 0.05). Finally, the high ODX/BR cases have a significantly smaller TFI than that obtained for the rest of cases (*p* < 0.01).

The primary conundrum in treatment and management of early stage estrogen receptor positive (ER+) breast cancer (BCa) is identifying which of these cancers are candidates for adjuvant chemotherapy and which patients will respond to hormonal therapy alone. ODX and other gene expression tests have allowed for distinguishing the more aggressive ER+ BCa requiring adjuvant chemotherapy from the less aggressive cancer benefiting from hormonal therapy alone. However these gene expression tests tend to be expensive, tissue destructive and require physical shipping of tissue blocks for the test to be done. Interestingly BCa grade in these tumors has been shown to be highly correlated with the ODX risk score^1^,^2^,^3^. Unfortunately studies have shown that Bloom Richarsdon (BR) grade determined by pathologists can be highly variable^4^. The three constituent categories within the BR grading system are mitotic index, tubule formation and nuclear pleomorphism. Tubule formation is defined as the percentage of cancer tissue that still contains normal tubules. According to Elston and Ellis guidelines^5^, tumor cell clusters with glandular formation are also counted (Fig. 1 shows some examples of tubule delineations for low and high risk BCa). Tubule scoring is determined by estimating tubule area and assigning to one of three categories: (i) >75%, (ii) between 10–75%, and (iii) <10%. However, this estimation is highly influenced by experience of the pathologist. Additionally, previous studies have shown the correlation between manually determined tubule score and ER+ breast cancer prognosis and ODX risk categories^6^,^7^.

Since histologic criteria (such as tubule, nuclei pleomorphism, and mitotic activity) are used in pathological grading systems, several works using automated extraction algorithms have been proposed to quantify such criteria^8^. Tubule detection has been previously addressed in the literature^9^,^10^,^11^. Typically these approaches focus on the identification of tubule lumen (see Fig. 1). Strategies focused on identifying tubules based off the lumen present a couple of challenges. Firstly the shape and size variability of the gland lumen makes accurate modeling of the tubules difficult. Secondly several structures, besides tubules, also contain lumen, i.e., blood vessels and other types of glands. Morphological operators have been used to connect proximal cancerous cells and generate blob structures^9^. These blobs were identified as tubules when they were found to be surrounding a white space or lumen. Another approach using the O’Callaghan neighborhood graph to impose structural constraints on lumen, allowed for identification of true lumen with an accuracy of 86%^10^. An accuracy of 89% was obtained in the classification task of low (tubular BR score 2 and 3) and high tubule formation (tubular BR score 1). A similar strategy, using *k*-means to identify lumen followed by a level set based segmentation approach enabled the identification of the surrounding nuclei layer^11^.

The deep neural network (DNN) is a deep learning architecture that comprises more than two hidden layers. In supervised classification settings, a DNN uses the backpropagation algorithm to update its internal weights according to the label of input exemplars^12^. Some applications of the DNNs in histological image analysis include the mitosis identification task^13^ and the localization of regions of interest in histological images^14^.

With the recent emergence of whole slide tissue scanning and digital pathology^15^,^16^,^17^ there has been substantial interest in developing automated computerized histologic predictors of tumor grade and outcome for several diseases including oropharyngeal squamous cell carcinoma^18^, prostate cancer^19^,^20^ and glioblastoma^21^. The correlation of computerized extracted features with breast cancer survival has also been explored. Beck *et al.*^22^ performed a comprehensive analysis of several automatically quantified morphological features and their relationship with breast cancer survival. The authors reported a strong association of automatically extracted stromal features with survival in a set of 576 H&E breast cancer tissue microarray (TMA) images. Tambasco *et al.*^23^ used fractal analysis to compute the morphological complexity of 379 pan-cytokeratin stained TMA images. A significant association of survival with the computed fractal dimension was found. The correlation of automated extracted features with Oncotype DX risk score and risk categories has been investigated in a couple of studies. Basavanhally *et al.*^24^ showed that nuclear graphs built using Delaunay triangulation and minimum spanning trees can be used to distinguish breast cancer images with low and high recurrence ODX scores (RS). The authors used 37 H&E stained images from a cohort of 17 patients at 20× magnification and obtained a mean accuracy of 84.15% in distinguishing samples with low and high RS. Also, the combination of computer extracted features from both H&E and CD34 IHC stained images in a cohort of 29 patients (9 with low RS, 11 intermediate RS and 9 with high RS)^25^ was shown to distinguish high and low ODX risk patients. The authors reported an average classifier accuracy of 91% for distinguishing high and low RS cases. Other studies have explored the association between manually identified pathological measurements (e.g. nuclei grade, mitotic index, tubule degree) and the Oncotype DX score. Both Flanagan *et al.*^6^ and Klein *et al.*^7^ used regression analysis to obtain a set of equations that predicts Oncotype DX score based on histological variables such as nuclei grade, mitotic index, tubule formation degree among others. After eliminating cases from the intermediate risk category, concordance between the ODX score and the estimated score (using the obtained equations) range from 96.9% to 100%.

The contributions of the work presented in this paper are twofold. Firstly we aim to evaluate a customized DNN for automatic quantification of tubules in whole slide images (WSI). Secondly we seek to evaluate whether tubule score automatically identified by the DNN is correlated with the risk categories determined by ODX in a cohort of 174 patients. Our approach comprises the following main steps. First, a blue ratio transform is used to detect nuclei candidates. Image patches, each containing a nucleus, are then extracted. These patches are manually labeled as containing a tubule or not. The patches are used to train a DNN classifier to identify tubule nuclei in WSI. After tubule nuclei identification, the ratio between tubule nuclei and overall number of nuclei is computed as a tubule formation indicator (TFI).

The rest of this paper is organized as follows: Section 2 describes the methodology used for training and testing the DNN tubule nuclei classifier. Section 3 presents the experimental design to study the correlation of the TFI with ODX risk categories. Section 4 describes the results of the statistical experiments and the distribution of the TFI for the ER+ BCa cases. Finally, in Section 5 we present the main conclusions of our work.

## Methodology

**Figure 2:**
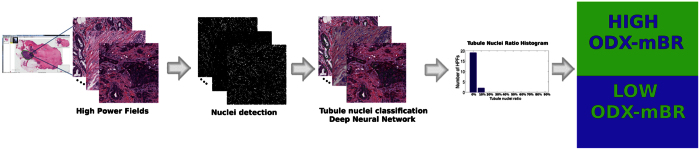
Overall diagram flow showing the steps to analyze the correlation of the tubule formation indicator with ODX score and BR grade. Several high power fields from a whole slide images are extracted. A nuclei detection method is then applied on each high power field. Each of the candidate nuclei is classified as tubule or not using a DNN classifier. Subsequently, the mean tubule nuclei ratio to total number of nuclei per high power field for each whole slide image is computed and analyzed with respect to the corresponding ODX risk category and BR grade.

The whole methodology to use the automated TFI to study its correlation with ODX score and BR grading in WSI is presented in Fig. 2.

### Nuclei detection

First, an automated algorithm based on blue ratio transformation^26^ is used to detect nuclei. After computing the blue ratio transform, a global threshold computed by using Otsu’s method^27^ is used to obtain a binary image. Then, an opening operation is applied. The centroid of each connected component corresponds to the centroid of a nucleus candidate. The nuclei detection algorithm is a lightweight method that provides a nuclei rough estimation that was found to be representative of the true nuclei population in terms of the TFI, as shown by the experiments described in the supplementary information.

### Curating the Learning Set

**Figure 3:**
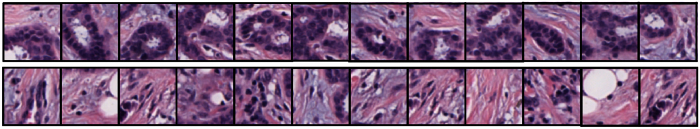
Examples of image patches used for training. Top Row: The tubule class. Bottom row: The non-tubule class. Each patch center corresponds to a nucleus candidate centroid.

An RGB patch is extracted (size 64 × 64 at 20x magnification with a spatial resolution of approximately 0.5 *μm* per pixel) around the centroid of each candidate nuclei. This patch is labeled as either tubule or not, according to an annotation supplied by an expert pathologist (The expert breast pathologist annotation corresponds to a manual delineation of each tubule). These pathologist annotated patches are then used to train the DNN classifier. Exemplar RGB patches belonging to the tubule class and non-tubule class are presented in Fig. 3.

**Figure 4:**
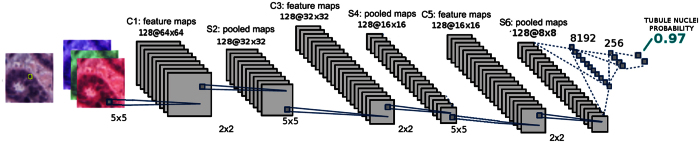
Deep learning architecture used to classify nuclei. A patch containing a nucleus feeds the deep neural network. The probability of the nucleus being part of a tubule is based on the output of the deep neural network classifier.

The DNN architecture is illustrated in Fig. 4 and is composed of three blocks: a convolution neural network (CNN), a Rectifier Linear Unit (ReLU) and a maximum pool (max pool) operator. Finally, two fully connected layers yield the probability representing the membership of the nucleus to the tubule class.

### Independent testing of the DNN classifier

During testing, the nuclei detection algorithm is used to identify candidate nuclear centroids. These patches then fed to the DNN, as shown in Fig. 4. This process enables the generation of their tubule class membership probability. If the probability is higher than 0.5, the patch is assigned to the tubule class.

The DNN performance was evaluated on a dataset with 61 high power fields that were extracted from 11 WSI. Whole tubule structures (including epidermis surrounding the lumen) had been previously annotated by an expert pathologist. A 5-fold cross validation setup was used, ensuring each fold was split at the patient level.

**Figure 5:**
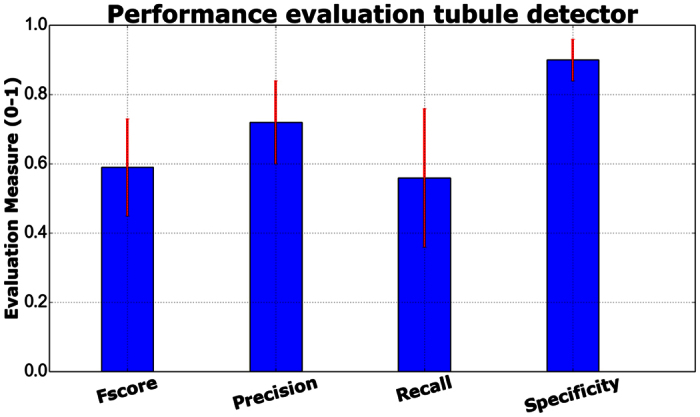
Performance evaluation measures for the tubule nuclei detection task in a 5-Fold cross validation setup and involving images extracted from *N* = 11 patients.

Evaluation measures (*F*_*score*_, precision, recall (sensitivity) and specificity for the tubule nuclei class^28^) were computed for each of the 5-folds. The average +/− standard deviation of the *F*_*score*_, precision, recall and specificity were: 0.59 ± 0.14, 0.72 ± 0.12, 0.56 ± 0.2 and 0.9 ± 0.06 respectively (see Fig. 5).

Observe that the recall for the tubule identification is lower than the specificity, indicating that a classification error is more likely for a tubule nuclei than for a non-tubule nuclei. Also, the variability of tubule sizes and shapes may explain the higher standard deviation obtained with the recall measure. Detailed results for each fold are presented in Table 1.

The detection results in Table 1 suggest that the tubule detector has a high specificity, a finding that might be caused by the unbalanced nature of the problem (there is a larger number of non-tubule nuclei as opposed to tubule nuclei in the BCa specimens). Also the tubule nuclei exhibit a substantially large inter-subject variation. The tubule nuclei samples used during training might not be adequate to capture all the variability observed in tubules from different patients.

## Experimental Design

### Data Description

A set of WSI extracted from 174 patients with ER+ BCa were used in this study. At most 50 high power fields per WSI were selected: the selected high power fields were those with the lower number of tubule nuclei ratio. This selection avoids high power fields with unusually large number of detected tubule nuclei (outliers). All of these high power fields were sampled from cancerous regions previously identified by an expert pathologist.

### Correlation with ODX risk groups via t-test analyses

After identifying the tubule nuclei the TFI was computed: the ratio between the tubule nuclei and the total number of nuclei. This TFI is evaluated as a potential risk predictor.

In order to compare the TFI with the risk associated to each BCa sample, the set was divided into a) High, b) Intermediate and c) Low risk categories according to the ODX score. Additionally, the BR grade is also used to define: d) The high ODX-high grade group (with both high ODX and BR score-HH), e) The low ODX-low grade group (with both low ODX and BR score-LL), f) All the BCa cases that don’t belong to the HH group (HHc group) and g) All the BCa cases that don’t belong to the LL group (LLc group). The dataset categorization is indicated in Table 2.

The t-test statistical analysis was applied to compare the distribution of the automated TFI with the high, intermediate and low ODX risk groups as well as the BCa cases with both a high ODX score and high grade and also cases with both low ODx score and low BR grade. The t-test for all the experiments was performed with equal mean and unequal variance hypothesis. Specifically, the t-test was applied to compare the different groups as described below:

- The high ODX group against the low ODX group
- The high ODX group against both the intermediate and low ODX group
- The low ODX group against both the high and intermediate ODX group
- The high ODX-high grade (HH Group) against the low ODX-low grade (LL group)
- The high ODX-high grade (HH Group) against all the other cases (HHc group) and
- The low ODX-high grade (LL group) against all the other cases (LLc group)

### Correlation with ODX risk groups via ROC analysis

The risk prediction capability of the TFI was also evaluated using a Receiver Operating Curve (ROC). For doing so, the binary classification task was based solely in the tubule nuclei ratio: each WSI with a mean tubule ratio above a particular threshold is classified as low ODX. By varying the threshold from [0, 1] is possible to generate the ROC curve. In this particular experiment the goal was to distinguish the HH and LL categories (see Table 2).

## Results

### Correlation with ODX and BR risk categories via t-test analyses

**Figure 6:**
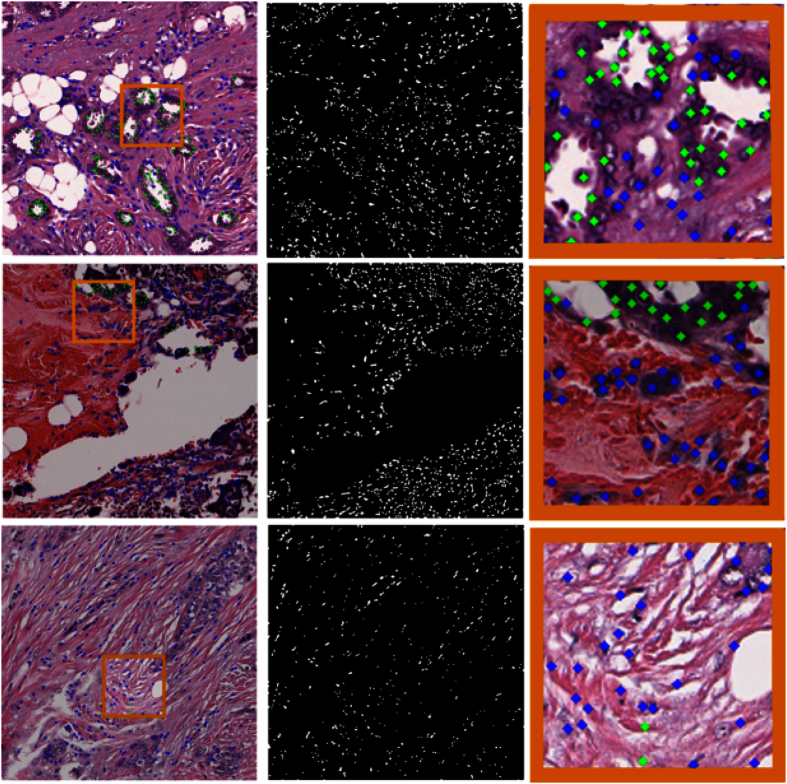
Tubule nuclei identification process for high power fields extracted from low ODX (top row), intermediate ODX (middle row) and high ODX (bottom row) breast cancers. In the first column, the high power field at x20 magnification is depicted. In the second column, the resulting mask showing the nuclei centroids after the nuclei detection process is presented. The third column shows the DNN classification of each nucleus either as a tubule nucleus (green dot) or a non-tubule nucleus (blue dots). Each image in the right column corresponds to a close up in the selected region (orange rectangle) depicted in the left most column. For the low ODX high power field, a significant number of tubule nuclei are identified. Observe also that some false negatives are not uncommon in the nuclei surrounding the tubule lumen. On the other hand, the high and intermediate cases have a substantially lower number of tubule nuclei. Some false positive (false tubule nuclei) errors are also visible in the right most column.

The DNN classifier was applied to the 174 WSI previously described. Qualitative results for high, intermediate and low ODX cases can be seen in the Fig. 6. The significant t-test results for the comparison between the risk groups is presented in Table 3.

**Figure 7:**
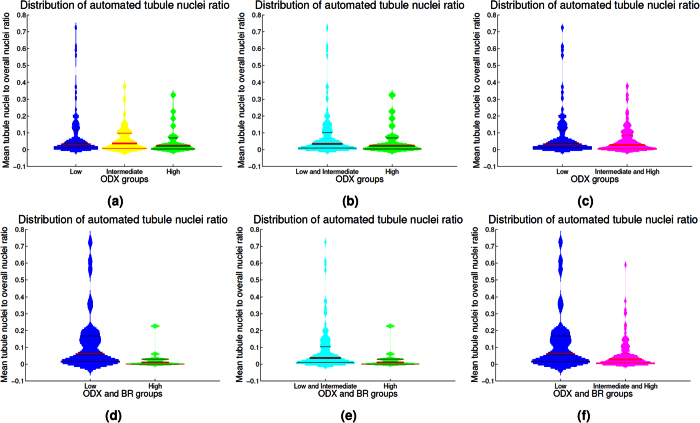
Violin plots depicting the mean tubule nuclei ratio in high power fields extracted from the different ODX risk groups. The histogram associated to each violin plot is smoothed using a normal kernel. Red lines in the violin plot show the location of the lower quartile (*q*_1_), the median and the upper quartile (*q*_3_). Low (blue), intermediate (yellow) and high (green) ODX groups are shown in the top row (**a**). The distribution of the low and intermediate groups (cyan) against the high ODX group is presented in (**b**). The low group against the intermediate and high ODX groups (magenta) are presented in (**c**). The distribution for the groups with low ODX-low grade and high ODX-high grade are depicted in (**d**). High ODX-high grade against all the other BCa cases and low ODX-low grade against all the other BCa cases are presented in (**e**) and (**f**) respectively.

When observing the group distribution according to ODX score, it is difficult to distinguish between low and high ODX groups. However, when combined ODX and BR groups are analyzed, the high and low risk groups show different distributions as shown in Fig. 7.

Results in Fig. 7 reveal that the automated TFI is significantly different for the groups that have low ODX-low grade and high ODX-high grade. The HH group had a mean tubule nuclei ratio per high power field of 0.029. In contrast, the LL group had a mean tubule nuclei ratio of 0.126. The two groups are significantly different (*p* < 0.01 with 95% CI [0.04, 0.16]). The differences in the TFI is still significant when we compare the HH group against the BCa cases that did not belong to this group (*p* < 0.01 with 95% CI[0.013, 0.085]). The mean for non HH cases was 0.078. Finally, the difference in the average TFI value was also significant when comparing the LL group with the BCa cases outside this group (*p* < 0.05 with 95% CI[0.014, 0.12]). The cases that did not belong to the LL group had a mean tubule nuclei ratio of 0.057.

### Correlation with ODX and BR risk categories via ROC curve

**Figure 8:**
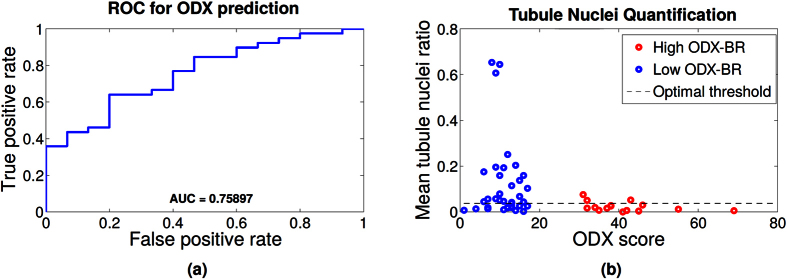
(**a**) Receiver operating characteristic (ROC) curve for the prediction of low ODX using only the tubule nuclei ratio feature. (**b**) Mean automated tubule nuclei ratio for each whole slide image. The high (red) and low (blue) ODX score groups are depicted. The x-axis represents the underlying ODX score of each sample. The y-axis represents the tubule nuclei ratio. Observe that the high ODX image have a low tubular density. A high tubule nuclei ratio is very likely associated with a low ODX image. Optimal threshold obtained for the ROC curve (threshold at which the ROC curve is closest to point [0, 1]) is also shown.

The distribution of the histologic images (ODX score vs tubule nuclei ratio) for the HH and LL groups is shown in the left column of Fig. 8. While a low mean tubule nuclei ratio appears to require additional analysis to determine its risk category, it is observed that a WSI with a high tubule nuclei ratio is very likely to be member of the low ODX risk category.

The Receiver Operating Curve (ROC) for the binary classification task using only mean tubule nuclei ratio for each WSI is presented in the right column of Fig. 8. The WSI with a mean tubule ratio above the threshold is classified as low ODX. The ROC curve shows that the tubule nuclei ratio yields an area under the curve (AUC) of 0.76 in distinguishing the low ODX-low grade from the high ODx-high grade categories.

### Concluding Remarks

In this paper we rigorously investigated the problem of objectively computing the tubule nuclei ratio, a potential computational histologic image biomarker of disease risk and aggressiveness in ER+ BCa. To evaluate whether automatically TFI was associated with the risk category determined by the Oncotype DX test, a deep learning classifier was developed to automatically identify tubules based off the surrounding nuclei. The automatically determined TFI was then evaluated in terms of its ability to distinguish the low and high ODX risk categories and cases with different permutations of ODX risk and grade. On a cohort of 174 WSI, the TFI was found to be significantly different for the BCa cases with low ODX-low grade and high ODX-high grade. When comparing the high ODX-high grade group with all the other BCa cases, the TFI was still significantly lower. Likewise, the calculated tubule quantification measure was larger in the BCa cases with low ODX-low grade compared to the remaining BCa cases.

The automated TFI appears to have a slightly weaker correlation with ODX risk categories than other previously investigated computerized image features such as nuclear architecture^24^. However it has been previously shown that using a combination of automated features (even extracted from differently stained samples from the same patient), might increase the ability to predict the corresponding ODX risk category^25^. Hence, developing strategies to integrate information from predictors that use different histological features (e.g. nuclear architecture, mitotic count, tubule density) will be a future research endeavor.

Automated tubule quantification could be potentially useful in streamlining clinical pathology workflows. The automated quantification aims to standardize the breast cancer grading and risk assessment process and reduce inter-reader variability. Our newly presented method was evaluated within manually selected cancerous regions. However, automatic delineation of regions of diagnostic interest is an open research problem^14^. Future work will focus on improving the tubule detector performance, validating our approach on larger test cohorts and incorporating automatic region of interest selection methods.

## Additional Information

**How to cite this article**: Romo-Bucheli, D. *et al.* Automated Tubule Nuclei Quantification and Correlation with Oncotype DX risk categories in ER+ Breast Cancer Whole Slide Images. *Sci. Rep.* **6**, 32706; doi: 10.1038/srep32706 (2016).

## Supplementary Material

Supplementary Information

## Figures and Tables

**Figure 1:**
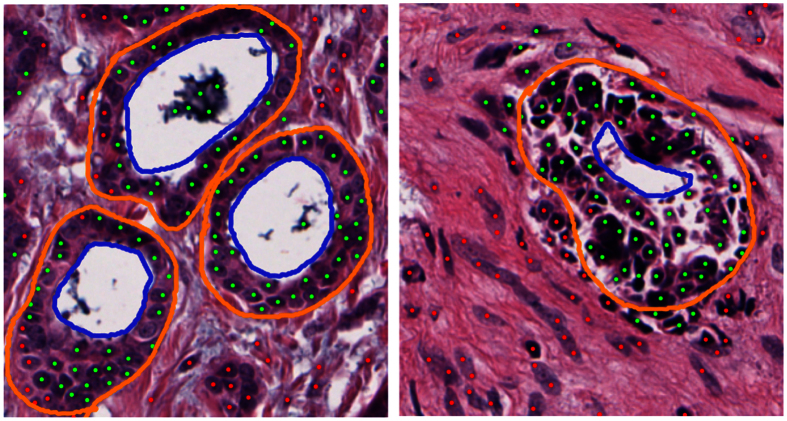
Breast Cancer tissue showing (left) high and (right) low tubule formation. Lumen is delineated by blue lines. Tubules are delineated by orange lines, where nuclei inside these boundaries represent the tubule nuclei used in our approach. Green and red dots correspond to nuclei candidates classified as tubule or non-tubule nuclei by our DNN classifier. In the high ODX (right) image, the cells have lost their capacity to form tubules with a rounded lumen.

**Table 1:** 5-Fold validation results for the tubule detection across *N* = 11 patients.

	*F-score*	*Precision*	*Recall (Sensitivity*)	*Specificity*
Fold 1	0.34	0.89	0.21	0.98
Fold 2	0.7	0.73	0.67	0.9
Fold 3	0.71	0.81	0.63	0.93
Fold 4	0.64	0.59	0.7	0.84
Fold 5	0.59	0.59	0.59	0.83
Average	0.59 ± 0.14	0.72 ± 0.12	0.56 ± 0.2	0.9 ± 0.06

The F-score, precision, recall and specificity for the tubule classification are shown. The final row presents the average and standard deviation for each of the four measures across all *N* = 11 patients and across the 5 folds.

**Table 2:** ODX score and BR grading rules used to split the dataset into high, intermediate and low ODX categories.

*BCa Groups*	*Description*	*Number of cases*
High ODX	*ODX* >30	24
Low ODX	*ODX* <18	95
Intermediate ODX	18≤ *ODX* ≤30	55
High ODX-high grade (HH)	Both *ODX* >30 and *BR* >7	15
Low *ODX*-low grade (LL)	Both *ODX* <18 and *BR* <6	42
HHc group	All BCa cases that do not belong to HH group	159
LLc group	All BCa cases that do not belong to LL group	132

The corresponding number of cases for each group is presented in the last column.

**Table 3:** Statistical comparison of the deep learning tubule classifier in distinguishing different risk groups.

Risk group comparison	p-values (Unequal variance)
H vs L	0.1633
H vs L and I	0.2731
H and I vs L	0.0998
**HH vs LL**	**0.0021**
**LLc vs LL**	**0.0145**
**HH vs HHc**	**0.0097**

Note that statistically significant differences were only observed for 3 of the 6 comparative experiments performed.
