# Supplementary material for: Automated Tubule Nuclei Quantification and Correlation with Oncotype DX risk categories in ER+ Breast Cancer Whole Slide Images
Source: Sci Rep. 2016 Sep 7;6:32706. doi: 10.1038/srep32706 (PMC5013328; doi:10.1038/srep32706)
Supplement: Supplementary Information [file srep32706-s1.pdf]

Supplementary File
Automated Tubule Nuclei Quantification and Correlation with Oncotype DX risk categories in ER+ Breast Cancer Whole Slide Images

David Romo Bucheli[1], Andrew Janowczyk[3], Hannah Gilmore[2], Eduardo Romero[1], and Anant Madabhushi[3*]

[1]Universidad Nacional de Colombia, Engineering Faculty, Bogota D.C, Colombia
[2]University Hospitals, School of Medicine, Cleveland, OH, USA
[3]Case Western Reserve University, Biomedical Engineering department, Cleveland, OH, USA
[*]anant.madabhushi@case.edu

In this supplementary file we present a experiment to evaluate the performance of the nuclei candidate detection method in the tubule nuclei detection task. A second experiment to measure the effect of the automated nuclei detection error in the computation of the tubule formation indicator (TFI) is also presented.

Experiment 1: Automatic tubule nuclei detection performance

Ten (10) images (high power fields-HPFs)from the tubule training dataset were selected and their nuclei were manually detected. To evaluate the performance of the automate nuclei detector, we compute the f-score measure in the tubule nuclei detection task for each image.
The F_{score} measure is computed using the following equation:

$$F_{score} = \frac{2 \cdot TP}{2 \cdot TP + FP + FN}$$

where TP corresponds to the number of true positive nuclei, FP is the number of false positives and FN is the number of false negative nuclei.
A candidate nucleus (obtained using the blueratio based nuclei candidate algorithm) is defined as true positive if it is within 10 pixels of a ground-truth nucleus. Otherwise it is counted as a false positive. Ground-truth nucleus that

are farther than 10 pixels from any candidate nucleus are counted as false negatives.
Results are presented in the following images. At the left we have the estimated nuclei detection for each HPF, while at the right we have the manually annotated image. Tubule nuclei are represented by a green circle.

Results for HPF 1

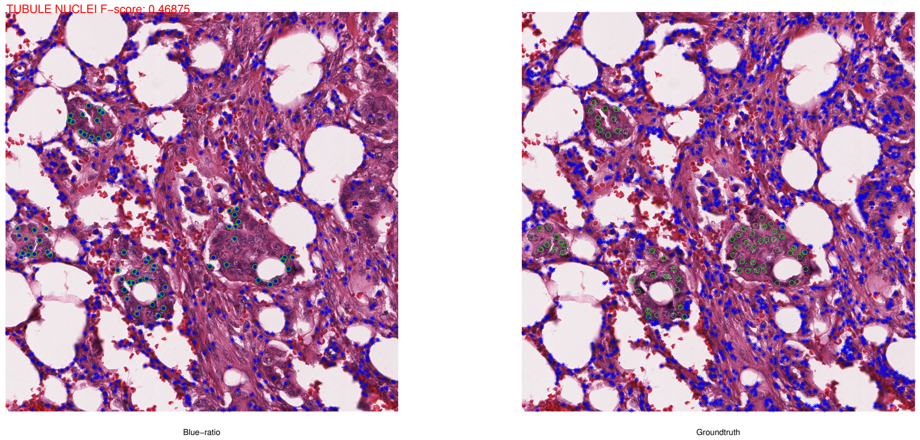

Results for HPF 2

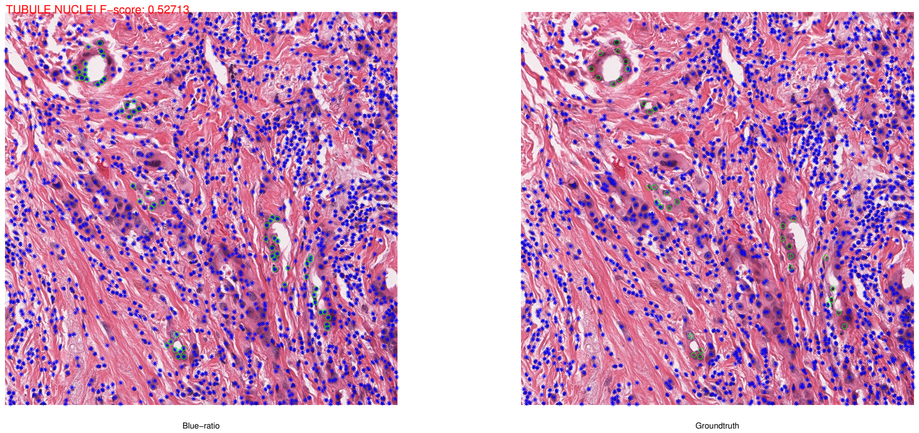

Results for HPF 3

TUBULE NUCLEI F−score: 0.62155

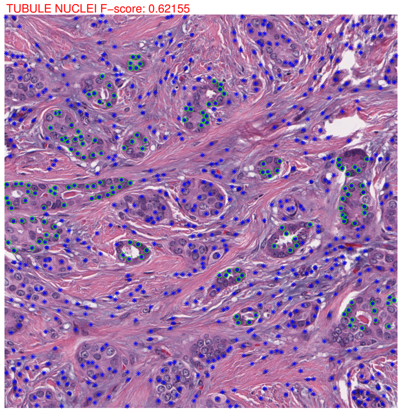

Blue−ratio

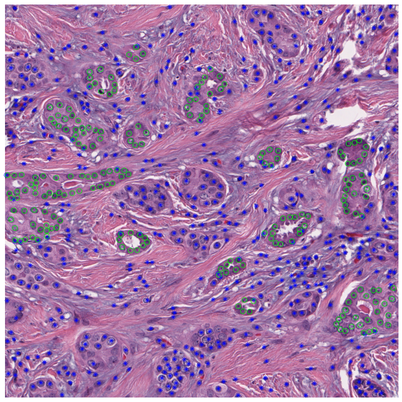

Groundtruth

Results for HPF 4

TUBULE NUCLEI F−score: 0.62538

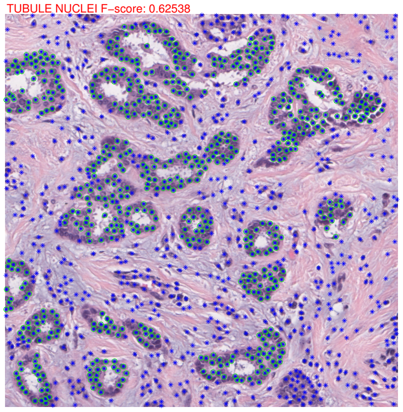

Blue−ratio

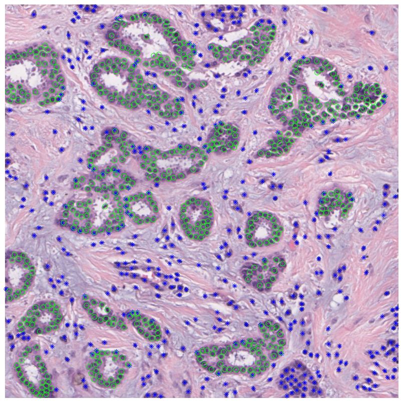

Groundtruth

Results for HPF 5

TUBULE NUCLEI F–score: 0.62169

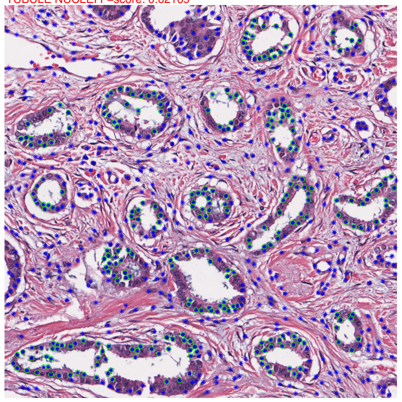

Blue–ratio

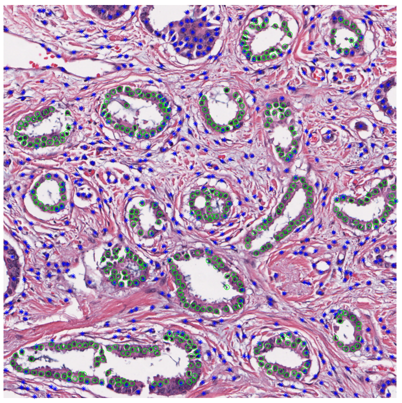

Groundtruth

Results for HPF 6

TUBULE NUCLEI F–score: 0.63028

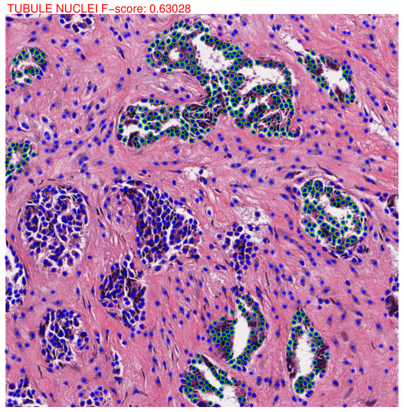

Blue–ratio

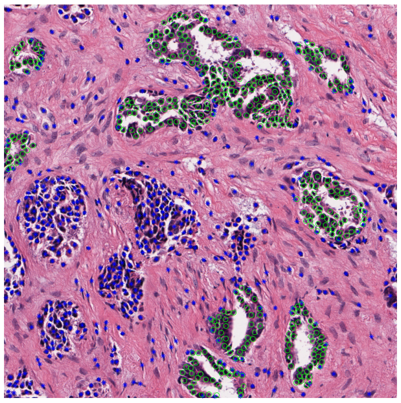

Groundtruth

Results for HPF 7

TUBULE NUCLEI F–score: 0.51831

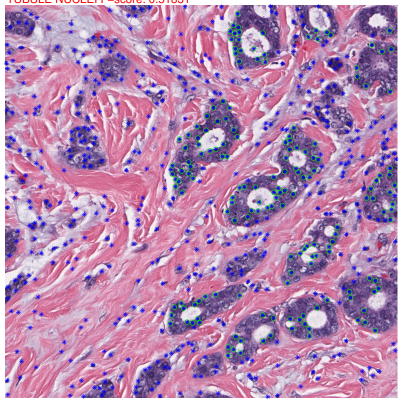

Blue–ratio

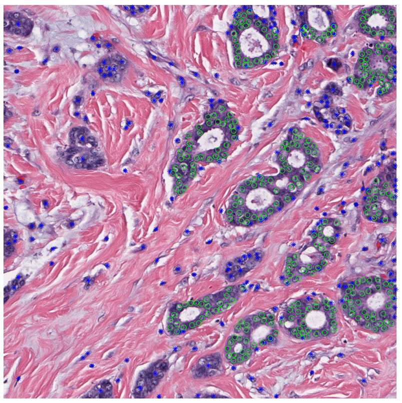

Groundtruth

Results for HPF 8

TUBULE NUCLEI F–score: 0.61317

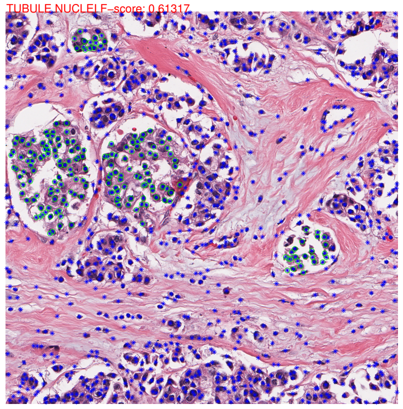

Blue–ratio

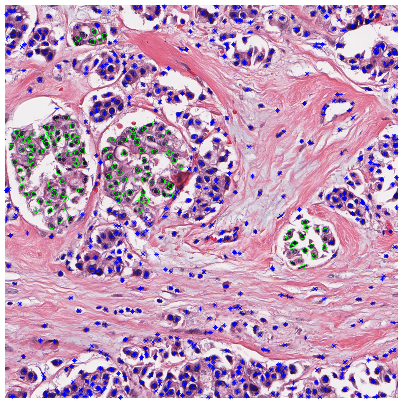

Groundtruth

Results for HPF 9

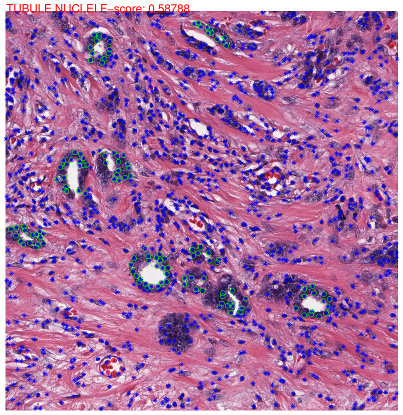

Blue–ratio

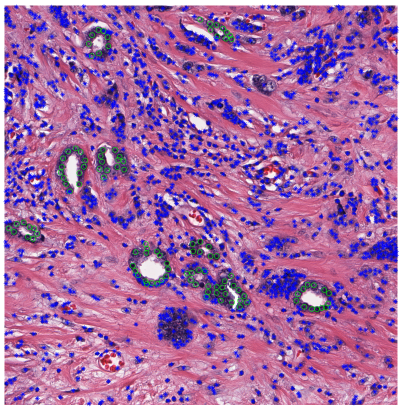

Groundtruth

Results for HPF 10

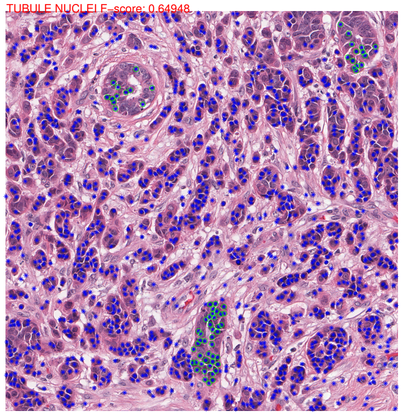

Blue–ratio

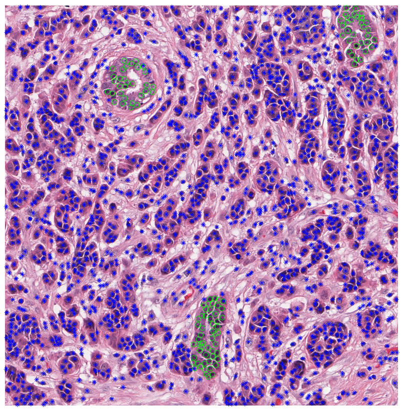

Groundtruth

The mean F-score for the 10 selected images is: 0.59

Experiment 2: Estimated TFI vs real TFI

In this experiment we use the same 10 images that we use in the previous experiment. Using the automated candidates nuclei and the pathologist manual annotations, we compute the real (based on ground-truth nuclei) and estimated (based on nuclei detected by the blue-ratio transform based algorithm) tubule formation indicator for each high power field. Results are summarized in the following graph:

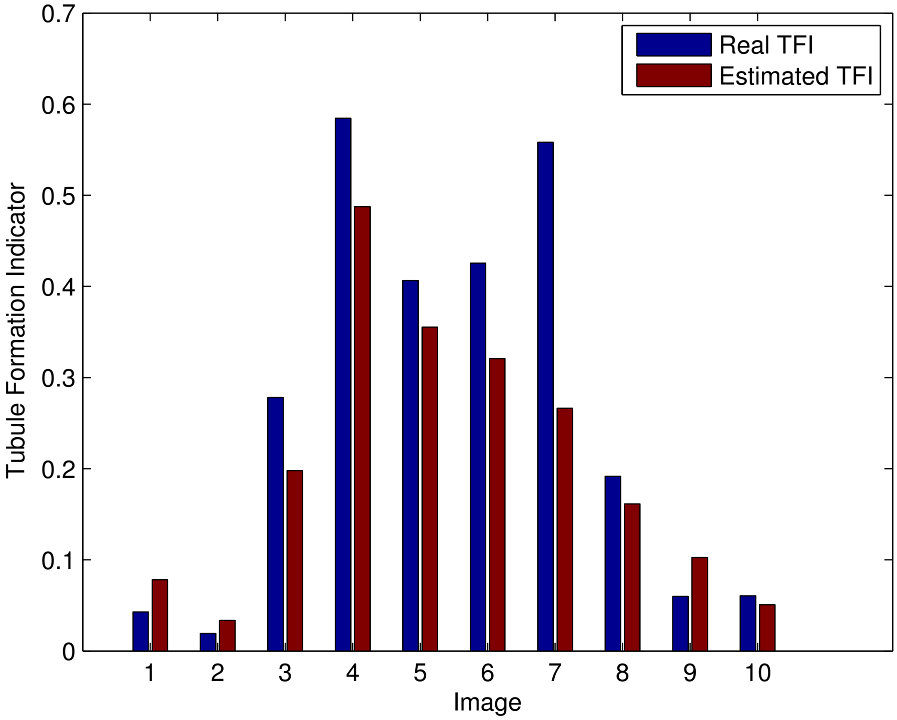

Results show that the nuclei candidate algorithm accuracy does affect the computation of the TFI. However the tendency generally holds, and HPFs with high/low real TFI usually have high/low estimated TFI. A median absolute error (MdAE) of 4.7% was obtained for the estimated TFI when using the automated nuclei detection algorithm.
